# Acute Liver Failure in Hemophagocytic Lymphohistiocytosis Secondary to Metastatic Renal Cell Carcinoma: A Diagnostic Dilemma

**DOI:** 10.7759/cureus.23455

**Published:** 2022-03-24

**Authors:** Mahir Qureshi, Andrew Alabd, Eric Behling, Roland Schwarting, Kathryn Haroldson

**Affiliations:** 1 Department of Medicine, Cooper Medical School of Rowan University, Camden, USA; 2 Department of Medicine, Cooper University Hospital, Camden, USA; 3 Department of Pathology, Cooper University Hospital, Camden, USA

**Keywords:** cytopenia, renal cell carcinoma, liver failure, malignancy, hemophagocytic lymphohistiocytosis

## Abstract

Hemophagocytic lymphohistiocytosis (HLH) is an aggressive hematologic disorder involving hyperstimulation of immune responses and severe inflammation. HLH has been well documented in lymphoid cancers and leukemias, but more rarely in solid tumors. The non-specific clinical characteristics of HLH can cause a diagnostic dilemma and delay in proper treatment, resulting in poor outcomes. We present a case of a patient with metastatic renal cell carcinoma who developed unexplained acute liver failure and was later found to have HLH. This case highlights the importance of including this syndrome on the differential diagnosis for acute liver failure of indeterminate cause and cytopenia in the setting of malignancy to facilitate proper timely treatment to improve outcomes and increase odds of survival.

## Introduction

Hemophagocytic lymphohistiocytosis (HLH) is characterized by an aggressive, unregulated, and often life-threatening hyperactivation of immune responses involving histiocytes and T cells with impaired function of natural killer cells [1]. HLH is most frequently observed in infants and young children, but younger and older adults can be affected as well [2-4]. Historically, HLH is distinguished into primary and secondary forms; however, this classification is often controversial, because the etiologies of both can be similar and, in some cases, identical. According to the recommendations from the North American Consortium for Histiocytosis, HLH is better characterized into four distinct categories: HLH syndrome, HLH disease, HLH disease mimics, and macrophage activation syndrome (MAS) [5].

Two methods are documented in the literature as effective in diagnosing HLH - the HLH-2004 diagnostic criteria and the H-Score [6-7]. For the HLH-2004 diagnostic criteria, there are two methods of diagnosing HLH: a molecular diagnosis of an HLH-associated gene, or a clinical diagnosis of five out of the eight features based upon the criteria recommended by the Histocyte Society. These criteria include fever ≥38.5 degrees Celsius, splenomegaly, peripheral blood cytopenia, with at least two of the following: hemoglobin <9 g/dL, platelets <100,000/microL, absolute neutrophil count <1000/microL, hypertriglyceridemia (fasting triglycerides >265 mg/dL) and/or hypofibrinogenemia (fibrinogen <150 mg/dL), hemophagocytosis in the bone marrow, spleen, lymph node or liver, low or absent NK cell activity, or ferritin >500 ng/mL. The H-Score predicts the likelihood of having HLH due to factors such as immunosuppression, temperature, organomegaly, number of cytopenias, ferritin, triglyceride levels, fibrinogen levels, aspartate aminotransferase (AST), and hemophagocytosis features on bone marrow aspirate [7].

Our case presents a patient who developed liver failure in the setting of HLH with concurrent renal cell carcinoma. Liver dysfunction is a common complication of HLH, but the relationship between HLH and liver dysfunction is not well established in the literature. There are also few reports in the literature of HLH presenting as acute liver failure in adults. However, one case report showed an adult patient who presented with symptoms of liver dysfunction with later workup showing evidence of HLH, but without concurrent malignancy [8]. Another case report discussed three middle-aged women with seemingly no remarkable medical history besides prior Epstein-Barr Virus (EBV) infection who developed acute liver dysfunction in the setting of HLH [9]. Additionally, there have been minimal reports of HLH in adults with concurrent solid organ malignancy, but such cases have been documented in the pediatric population, primarily with lymphoid cancers and leukemias. To date, there have been two case reports on the development of HLH in the setting of renal cell carcinoma [10-11].

## Case presentation

A 51-year-old man with a medical history of metastatic renal carcinoma and diabetes mellitus presented with a three-week history of progressive fatigue, muscle aches, watery diarrhea, shortness of breath, and loss of appetite. Physical examination was remarkable for scleral icterus, jaundice, abdominal distention, and a liver spanning approximately 12 cm with flank dullness and a positive fluid wave.

The initial laboratory findings (Table 1) were notable for electrolyte abnormalities, anion gap metabolic acidosis, and abnormal liver function tests. Lipase levels were within normal limits. Hepatitis A, B, and C were all negative.

**Table 1:** Laboratory test results CMV: Cytomegalovirus; EBV: Epstein-Barr Virus; HSV: Herpes Simplex Virus The superscript * denotes an abnormal laboratory value.

Laboratory Test	Results	Normal Range
Sodium (Na+)	127 mmol/L*	136-145 mmol/L
Potassium (K+)	5.3 mmol/L*	3.5-5.0 mmol/L
Chloride (Cl-)	94 mmol/L*	96-108 mmol/L
Bicarbonate (HCO3-)	14 mmol/L*	22-28 mmol/L
Calcium (Ca2+)	11.4 mg/dL*	8.5-10.5 mg/dL
Anion Gap	19 mmol/L*	7-16 mmol/L
Lactate	5.2 mmol/L*	0.5-2.2 mmol/L
Hemoglobin	8.4 g/dL*	14-18 g/dL
International Normalized Ratio (INR)	1.6*	0.8-1.2
Prothrombin Time (PT)	19.1 seconds*	9.9-13.5 seconds
Partial Thromboplastin Time (PTT)	34.7 seconds	27.5-38.3 seconds
Aspartate Aminotransferase (AST)	344 U/L*	10-35 U/L
Alanine Transaminase (ALT)	217 U/L*	6-45 U/L
Alkaline Phosphatase (ALP)	302 U/L*	39-117 U/L
Total Bilirubin	3.4 mg/dL*	0.1-1.0 mg/dL
Direct Bilirubin	2.5 mg/dL*	0.0-0.3 mg/dL
Lipase	31 U/L	16-63 U/L
Lactate Dehydrogenase (LDH)	2,356 U/L*	45-90 U/L
Ferritin		15-400 ng/mL
Hospital Day 2	4,879 ng/mL*	
Hospital Day 11	8,936 ng/mL*	
Ceruloplasmin	53 mg/dL*	18-36 mg/dL
Alpha-1 Antitrypsin	>300 mg/dL*	83-199 mg/dL
Hepatitis A	Negative	
Hepatitis B	Negative	
Hepatitis C	Negative	
CMV DNA, PCR	<200 IU/mL	<200 IU/mL
EBV Viral Capsid IgG	663 U/mL*	18.00-21.99 U/mL
EBV Viral Capsid IgM	<36 U/mL	36.00-43.99 U/mL
EBV Nuclear Antigen	141 U/mL*	18.00-21.99 U/mL
HSV-1	Not Detected	

**Figure 1:**
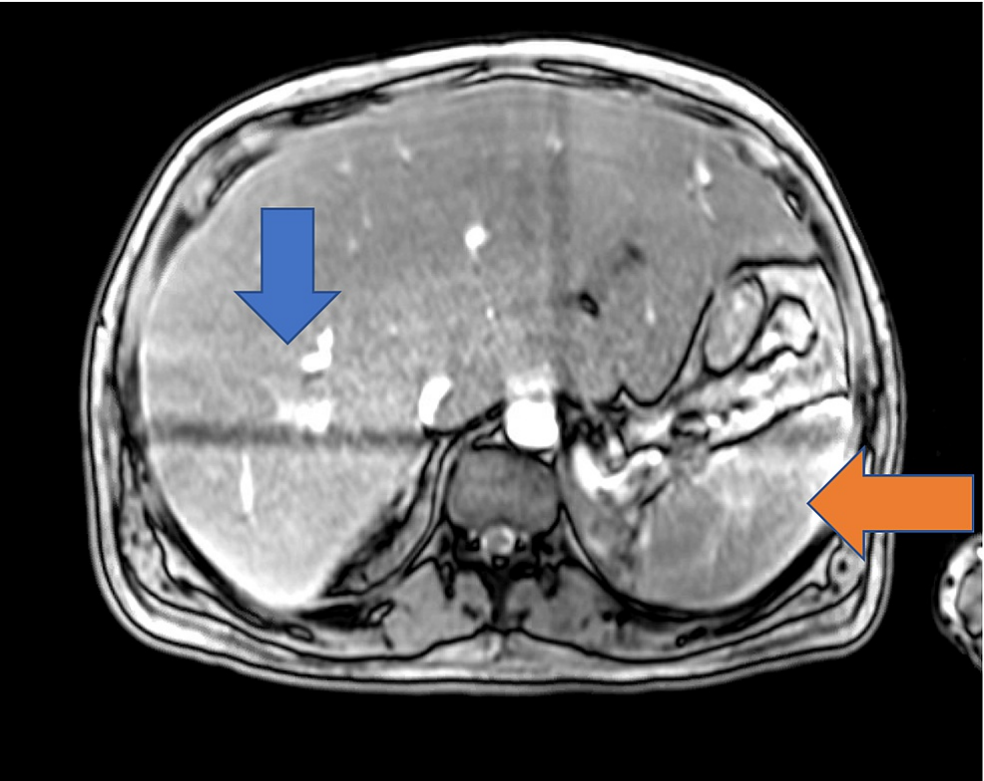
MRI of the liver with contrast Markedly enlarged liver with abnormal T2 signal (blue arrow) and heterogeneous enhancement with splenomegaly (orange arrow).

**Figure 2:**
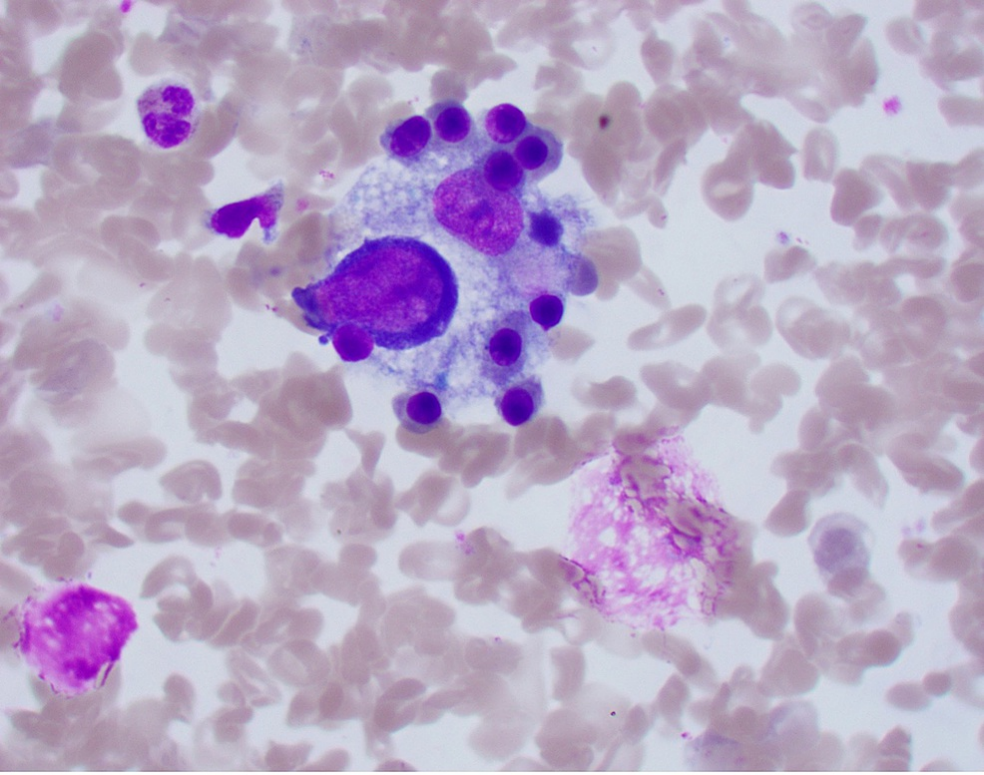
Bone marrow aspirate A large foamy macrophage engulfing numerous erythroid precursors seen within a bone marrow aspirate smear (Wright-Giemsa stain, original magnification x1000).

Chest X-ray did not show any evidence of acute cardiopulmonary disease, and a subsequent computed tomography (CT) scan of the chest was negative for pulmonary embolism. A CT scan of the abdomen and pelvis with IV contrast showed evidence of an enlarged liver with numerous foci of wedge-shaped and round hyper-attenuation with liver infarctions with two small right hepatic lesions and small ascites. Ultrasound of the right upper quadrant showed evidence of portal hypertension, splenomegaly, and biliary sludge. MRI of the liver with contrast showed heterogenous, geographic hypo-enhancement in the regions of abnormal T2 signal, and hepatic lesions were found in the posterior right hepatic lobe, right hepatic lobe, and lateral segment (Figure 1). Bone marrow aspirate smear demonstrated a foamy macrophage engulfing precursor erythroid cells (Figure 2).

On hospital day 2, further laboratory work showed an elevated ferritin and lactate dehydrogenase levels (Table 1). On hospital day 8, the hospital course was complicated by worsening respiratory status, as well as liver and kidney failure. The presence of liver lesions from metastatic renal cell carcinoma did not explain the acuity of the patient’s liver failure. He was scheduled for a liver biopsy to rule out infiltrative diseases of the liver, but he was unable to tolerate a liver biopsy. On hospital day 11, his ferritin continued to uptrend (Table 1) and a right upper quadrant with Doppler ultrasound was performed, which showed evidence of a new left portal vein thrombus. His overall illness continued to worsen, and his care was escalated to the intensive care unit on hospital day 12, where he was intubated and underwent renal replacement therapy. He was started on chemotherapy with etoposide and dexamethasone for presumed HLH.

Further laboratory examination was significant for anti-smooth muscle antibody, anti-nuclear antibody, and anti-mitochondrial antibody, all of which were negative. Cytomegalovirus (CMV), herpes simplex virus (HSV), and varicella-zoster virus (VZV) PCR were negative as well. EBV PCR showed evidence of a past EBV infection. Ceruloplasmin and Alpha-1 anti-trypsin were elevated (Table 1). Bone marrow biopsy also showed an increased number of cells exhibiting hemophagocytosis, which along with the laboratory findings confirmed the suspicion of HLH. The suspicion arose due to the evidence of splenomegaly on imaging, uptrend of ferritin, uptrend of and markedly elevated lactate dehydrogenase, hypertriglyceridemia, elevated international normalized ratio (INR), bicytopenia with anemia and thrombocytopenia, and uptrend of aspartate aminotransferase/alanine transaminase (AST/ALT). Subsequently, soluble CD25 and Gamma GT were found to be elevated (Table 1).

The patient’s clinical condition continued to worsen drastically and was further complicated by encephalopathy, shock, worsening cholestatic jaundice, and seizures. He was transitioned to hospice care, where he died surrounded by his loved ones.

## Discussion

HLH should be considered in the differential diagnosis of patients with acute liver failure of indeterminate cause and cytopenia in the setting of malignancy. HLH in the setting of renal cell carcinoma is exceptionally rare, with only two cases reported to date [10,11].

Our patient met six of the eight original HLH-2004 diagnostic criteria, which included fever, splenomegaly, peripheral blood bicytopenia, hypertriglyceridemia, ferritin >500 ng/mL, and hemophagocytosis in the bone marrow. Additionally, our patient had a high predictive value H-score of 228, signifying a 96-98% chance of having HLH. Our patient was on immunosuppression agents due to the known malignancy (18 points), hepatosplenomegaly (38 points), bicytopenia (24 points), ferritin levels above 6000 (50 points), AST above 30 (19 points), and hemophagocytosis features on bone marrow aspirate (35 points). Both diagnostic criteria, HLH-2004 and H-score, are accurate in the diagnosis of HLH in our patient.

The common viral causes of HLH include EBV, HIV, HSV, CMV, viral hepatitis, and influenza [12]. Our patient did not have any known active infections; however, he did show evidence of a prior EBV exposure in the past. With the lack of any known significant risk factors for developing HLH at presentation, it is possible that this syndrome could have been caused by the advanced metastatic process of the patient’s renal cell carcinoma.

A previous case report describes a patient with renal cell carcinoma who developed HLH and who exhibited marked improvement of symptoms and subsequent discharge after receiving high-dose steroid therapy [11]. There is also evidence highlighting the effectiveness of an eight-week induction regimen using corticosteroids and chemotherapy such as etoposide when the HLH is EBV-driven [13]. However, our patient did not improve after receiving dexamethasone and etoposide, which further supports the possibility of the malignancy being the driver for the development of HLH or perhaps being multifactorial in nature. In the second case report of the development of HLH, a patient with renal cell carcinoma on pazopanib therapy also had negative serology of the commonly recognized viral drivers of HLH but did have a history of systematic lupus erythematosus (SLE) without any signs of active disease [11]. There is evidence that SLE could be a potential risk factor for HLH; however, with no signs of active disease, there was more suspicion of HLH being caused by the malignancy [11,12]. Interestingly, to our knowledge, our patient did not have any significant medical history besides malignancy that could have potentially driven the onset of HLH.

One of the common complications of HLH is liver dysfunction, which is rarely seen as the presenting concern in adults. However, for our patient, the physical examination findings at presentation were of hepatic origin, such as scleral icterus, jaundice, and abdominal distention. The suspicion of HLH was raised when the workup for liver dysfunction initially yielded negative results. The etiology of this patient’s presentation was less likely to be the metastatic process of the patient’s renal cell carcinoma because of the other diagnostic findings of HLH found after further workup such as peripheral blood bicytopenia, hypertriglyceridemia, elevated ferritin, and hemophagocytosis in the bone marrow. Furthermore, the patient’s condition did not improve after the administration of etoposide and dexamethasone, which was shown to improve the cytokine storm caused by HLH in a previous case report [11].

Nevertheless, our case exemplifies the importance of keeping HLH in the differential diagnosis of a patient with a known or suspected malignancy with evidence of liver dysfunction of unknown origin and other pertinent features reported in the original HLH-2004 diagnostic criteria and the H-score.

## Conclusions

HLH presenting as acute liver failure in an adult with metastatic renal cell carcinoma is very rare and is likely under-diagnosed, especially due to its nonspecific clinical characteristics. Even though anecdotal evidence has shown high-dose steroids to be effective in HLH with known solid tumor malignancy, extensive further data are needed to assess the efficacy of treatment options. Given the high mortality of this disease process, it is imperative to keep the rare occurrence of HLH in the differential diagnosis for liver dysfunction of unknown cause.
